# Beyond Structural Neglect: Using Evidence to Strengthen Primary Health Care Systems, Policies and Programs for Aboriginal and Torres Strait Islander Men

**DOI:** 10.5694/mja2.70319

**Published:** 2026-10-16

**Authors:** Kootsy Canuto, Celina Gaweda, James Smith, Karla J. Canuto

**Affiliations:** ^1^ College of Medicine and Dentistry James Cook University Cairns Queensland Australia; ^2^ Rural and Remote Health NT, College of Medicine and Public Health Flinders University Darwin Northern Territory Australia

**Keywords:** health policy, health systems, Indigenous health, primary care

## Abstract

Despite decades of policy focus, Aboriginal and Torres Strait Islander men still face primary health care systems that are misaligned with their cultural and social needs, leading to low use. A strong body of evidence shows engagement improves when care is culturally safe, gender‐responsive and community‐led, yet these are inconsistently implemented. Short‐term funding and fragmented commissioning undermine continuity, workforce stability and holistic care. Reliance on pilot programs reflects risk aversion and limits reform. Redesigning commissioning to ensure long‐term funding, a stable workforce, stronger governance and learning‐focused evaluation is essential. Continued delay perpetuates inequity, making reform an ethical and political imperative.

## 1 Positionality Statement

This research is undertaken by a team with longstanding, collaborative commitment to Aboriginal and Torres Strait Islander health, with a particular focus on Indigenous men's health and wellbeing. Associate Professor Kootsy Canuto is a Wagadagam man from the Torres Strait and a nationally recognised leader in Aboriginal and Torres Strait Islander men's health research. His work centres Aboriginal and Torres Strait Islander men's experiences, including fatherhood, primary health care engagement and social and emotional wellbeing, and is embedded in strong, enduring community partnerships. Professor Karla Canuto is a proud Torres Strait Islander woman from the Naghir Tribe of the Kulkalgul clan, with ancestral ties to Mabuyag Island. Her identity, family responsibilities and commitment to Indigenous health underpin her research, which spans more than two decades and is grounded in Indigenous leadership, self‐determination and community authority. Celina Gaweda is a non‐Indigenous researcher of Polish descent who has worked in Aboriginal and Torres Strait Islander health research for over 5 years, focusing on co‐designed, community‐led approaches to men's health and wellbeing. Her work prioritises cultural safety, respectful engagement and local research capacity. Professor James A Smith is a non‐Indigenous Australian of British and Irish ancestry who has lived and worked for nearly two decades in the Northern Territory. His work in partnership with Aboriginal and Torres Strait Islander communities spans government, non‐government and academic settings, with a focus on applied men's health and health equity. Together, the team brings complementary perspectives and extensive experience in Aboriginal and Torres Strait Islander men's health. This work is guided by principles of relational accountability, cultural safety and a shared commitment to strengthening Indigenous‐led knowledge and practice. The CONSIDER reporting criteria checklist for health research involving Indigenous peoples was completed for this article and can be found in the Supporting Information S1 [1].

## 2 The Policy Paradox

Over the past two decades, Aboriginal and Torres Strait Islander men have consistently been identified as a priority population within national health policy frameworks. These men experience the highest burden of disease, the lowest life expectancy and the lowest rates of primary health care (PHC) utilisation of any major population subgroup in Australia [2, 3, 4]. The current life expectancy of Aboriginal and Torres Strait Islander males is 8.8 years less than non‐Indigenous males, and 4322 Aboriginal and Torres Strait Islander male deaths between 2015 and 2019 were deemed preventable with timely and effective health care [5]. Studies have shown that the underutilisation of primary health care services (PHCS) does not reflect their disengagement but systems and services that remain difficult to access and navigate, and inadequately resourced to meet culturally and gender‐specific needs and expectations [2, 6, 7].

These systemic barriers to PHC are well documented in national reports, academic literature and government strategies [8, 9, 10]. Despite this, progress has been modest. Successive initiatives have produced promising pilot programs, many indicating potential for sustained health impact [2, 11, 12]. However, few have been embedded or scaled in ways that produce durable and systemic change. Instead, policy responses continue to rely on time‐limited interventions operating at the margins of existing health systems, thus creating a paradox [13, 14].

Although evidence and effective practice are well established, structural reform remains limited. Aboriginal and Torres Strait Islander men continue to encounter PHCS poorly aligned with their social, cultural and practical realities [2, 3, 15]. Although these system shortfalls affect all Aboriginal and Torres Strait Islander populations, the health status of men has remained persistently low. Without addressing access barriers through policy, reform efforts are likely to reproduce suboptimal outcomes [2, 11, 12].

## 3 What the Evidence Shows

Despite an urgent need to invest more heavily in Aboriginal and Torres Strait Islander male health and wellbeing research [16], substantial evidence has established the factors influencing engagement with PHC. Qualitative studies consistently identify barriers including long waiting times, culturally inappropriate environments, limited availability of male practitioners, negative prior experiences and concerns about confidentiality and respect [2, 6, 17, 18]. Simultaneously, they demonstrate strong motivation for preventive care, such as adult health checks when services are accessible, culturally safe and relevant [6, 19, 20, 21].

Early qualitative work highlighted that men often delay care because services are experienced as uncomfortable, unwelcoming or misaligned with their priorities [2]. Subsequent studies confirm that feelings of shame, embarrassment and vulnerability are shaped by service environments rather than intrinsic attitudes [6, 20, 22]. Systematic reviews similarly demonstrate that limited progress in improving utilisation reflects structural shortcomings rather than individual behaviour [2, 19, 23]. Despite policy recognition of Aboriginal and Torres Strait Islander men as a priority population, relatively few rigorously evaluated engagement strategies have been embedded within routine practice [2, 12, 24].

Research by Brown and colleagues further demonstrates that Aboriginal men's health disadvantage is mediated by chronic psychosocial stress, depression and the interconnected burden of cardiovascular and mental illness [25]. These conditions are shaped by socio‐economic marginalisation, cumulative stress and cultural disconnection, rather than individual lifestyle choices. Brown's work also highlights the importance of culturally specific assessment tools for identifying distress and supporting effective engagement [25, 26, 27]. Earlier research similarly emphasised that improving men's health requires recognition of illness, culturally acceptable services and sustained community engagement, rather than reliance on conventional general practice models alone [7, 28, 29].

Services demonstrating sustained client engagement in PHC are characterised by strong Aboriginal and Torres Strait Islander leadership, relational trust, flexible delivery models and consistent resourcing [11, 20, 30, 31]. Where these features are present, engagement improves. Where they are absent, it declines. Current evidence challenges deficit‐based narratives that frame Aboriginal and Torres Strait Islander men as ‘hard to reach’, demonstrating that men engage when health systems adapt to their needs [2, 12, 19].

## 4 Why Reform Repeatedly Falls Short

If the evidence base is undeniable, why has reform been so limited? A central constraint lies in the way PHC initiatives are funded and governed. Aboriginal Community Controlled Health Organisations (ACCHOs) are PHCS governed and run by the local Aboriginal community, playing a vital role in delivering holistic, comprehensive and culturally appropriate care [20]. State and territory‐run services also provide PHC to Aboriginal and Torres Strait Islander people; however, they are generally less socially and culturally accessible. As a result, ACCHOs remain the preferred option for First Nations people [32]. Although ACCHOs and mainstream services do not compete for the same pool of funding, ACCHO's are critically underfunded, receiving significantly less despite servicing communities with disproportionately higher health needs than non‐Indigenous populations [33].

Federal investment in Indigenous health remains heavily reliant on short‐term, competitive grant cycles primarily administered through the Commonwealth Department of Health, Ageing and Disability, state and territory governments and Primary Health Networks (PHN) [2, 11, 12, 13, 14, 32]. Programs are commonly funded for 2–3 years, with limited provision for consolidation, workforce development and retention or long‐term monitoring and evaluation. Grant funding also requires substantial resources for applications, reporting and compliance. This administrative burden hinders service development and community engagement and favours organisations with greater grant‐writing capacity rather than those with the strongest local relationships [14, 32, 34]. In turn, this exacerbates health and social inequities experienced by Aboriginal and Torres Strait Islander men.

Workforce instability further compounds these problems. Short funding cycles limit the ability to recruit, retain and develop Aboriginal and Torres Strait Islander health professionals and facilitators. High staff turnover disrupts continuity of care, weakens trust, affects institutional memory and erodes community confidence in service reliability [11, 30, 32, 35, 36, 37]. Workforce stability is also influenced by access to quality education and training, effective governance and workforce conditions [37, 38].

Fragmentation across funding streams also undermines reform. Separate programs addressing chronic disease, mental health, substance use, family wellbeing and social support frequently operate in parallel, with limited cross‐program coordination. Men are expected to navigate complex service landscapes (particularly in mainstream services) that do not reflect the culturally appropriate and holistic nature of Aboriginal and Torres Strait Islander health and wellbeing concepts [2, 14, 39, 40, 41]. Monitoring and evaluation frameworks, often developed without meaningful Indigenous input, prioritise quantifiable indicators over community‐defined outcomes, limiting learning, quality improvement and system‐wide scaling [2, 11, 12, 39, 42]. Together, these structural features produce a system that excels in generating innovative pilot programs but struggles to deliver end‐to‐end service reforms in a sustained way [11, 14, 32, 42].

## 5 System Reorientation in Practice

More than two decades of policy documents have called for culturally responsive, community‐led and integrated primary care [8, 9, 10, 15]. The practical application of these commitments is now also well established in the evidence base. Sustained engagement of Aboriginal and Torres Strait Islander peoples in PHCS is evident when governance frameworks embed Aboriginal and Torres Strait Islander leadership and local accountability within decision‐making processes [11, 20, 30]. These structures shape service priorities, workforce investment and resource allocation. Services are better aligned with community‐defined needs when governance authority is meaningful and stable; where it is symbolic, engagement efforts remain fragile [15, 23].

Gender‐responsive service design sits at the core of strong Aboriginal and Torres Strait Islander governance. Dedicated men's clinics, Aboriginal and Torres Strait Islander male health workers, and culturally safe spaces improve engagement by normalising culturally safe care [3, 6, 19]. However, continued dependence on short‐term funding and individual champions reflects the marginal status of Aboriginal and Torres Strait Islander male health workers as decision‐makers and leaders within prevailing service standards, leaving them with their hands tied and unable to implement and progress solutions they know would improve access and utilisation of PHC [13, 34].

Although not unique to Aboriginal and Torres Strait Islander men, effective health care models demonstrate that clinical care cannot be separated from social, cultural and wellbeing supports. Integration is not an optional enhancement but a functional requirement for addressing interconnected health burdens and the holistic nature of social and emotional wellbeing [21, 39, 40, 43]. Despite its strengths, Australia's health system is fragmented across the primary, secondary and tertiary care continuum [44]. Generally, mainstream commissioning models used to fund health programs are typically assessed around specific health needs or domains. The process is ongoing, administratively intensive and often involves data‐heavy reporting requirements, with monitoring and evaluation frameworks inadequately aligned with holistic models of care or the pursuit of long‐term health and wellbeing outcomes for Aboriginal and Torres Strait Islander people [41]. Together, these factors undermine integrated practice. Funding timeframes, along with monitoring and evaluation frameworks, mediate how key elements of programs evolve. Frameworks that privilege Indigenous knowledges and support co‐design, participatory learning and continuous quality improvement enable services to adapt to community feedback and emerging needs [13, 41]. By contrast, evaluation frameworks, focused on metric‐based key performance indicators, rather than meaningful health outcomes tend to lock in existing practices and discourage innovation [40, 44]. As such, evaluation processes play a critical role in determining whether learning and collaboration are embedded in practice. The continued reliance on pilot programs for Aboriginal and Torres Strait Islander men, in the absence of program scaling and broader structural reforms, demonstrates the need for a more coherent policy architecture [13, 32]. The principles underlying effective PHC practice are well established. Limited uptake reflects not a deficit of evidence, but a lack of institutional commitment.

## 6 More Cohesive Commissioning

One mechanism for advancing health system reform lies in redesigning PHN commissioning practices, specifically those relating to Aboriginal and Torres Strait Islander PHCS [2, 11, 12, 13, 32]. Current PHN contracts frequently emphasise short‐term activity targets and frequent reporting cycles, often based on contractual obligations imposed by the Australian Government. Improved commissioning would include minimum 5‐year funding horizons for ACCHOs and partner services delivering gender‐specific programs, with explicit provisions for workforce development, governance support and integrated service delivery [30, 33, 34].

Commissioning contracts could also mandate the inclusion of male Aboriginal and Torres Strait Islander health professionals as ongoing funded roles; link a proportion of payments to demonstrated continuity of engagement rather than service volume; and require co‐designed monitoring and evaluation frameworks focused on learning and quality improvement [2, 30, 40]. This approach would stabilise effective practice and reduce reliance on perpetual pilot funding while using existing Commonwealth commissioning mechanisms [9, 10, 14, 15, 43].

Action 4 in the Productivity Commission's report on Indigenous Evaluation Strategy provides a promising commitment to ensuring ‘agencies’ involved in the design, commissioning, management and delivery of evaluations impacting Aboriginal and Torres Strait Islander people possess the required capability, including relevant skills, experience and understanding [13]. In principle, this aligns closely with the priority reforms outlined in the 2020 National Agreement on Closing the Gap: (1) Formal partnerships and shared decision‐making; (2) Building the community‐controlled sector; (3) Transforming government organisations; and (4) Shared access to data and information at a regional level [45]. However, 6 years on, there has been slow progress to warrant true optimism.

## 7 Implications for Accountability

From a federal health policy perspective, the current approach represents poor value for public investment. Substantial resources have been directed towards short‐term initiatives that fail to generate system‐wide or long‐term health impact [11, 14, 32]. Delayed presentation, unmanaged chronic disease, avoidable hospitalisations and premature mortality generate long‐term costs that exceed the investment required for effective PHC reform [23, 46]. Preventive, culturally responsive services will be more cost‐effective than crisis‐driven care [10, 14, 31, 42, 44, 45, 46, 47].

Accountability therefore requires more than reporting on program outputs. It requires transparent assessments of the alignment of funding and governance arrangements with established evidence on what works in concert with evidence of meeting community needs [10, 32, 41]. Commonwealth agencies, PHNs and other funding providers each hold defined responsibilities in this process. Aligning funding cycles with workforce needs, embedding governance capacity within contracts and supporting integrated evaluation, are measurable policy choices. Continuous failure to address these deficiencies warrants scrutiny [13, 14, 32, 44].

## 8 From Evidence to Action

The evidence regarding Aboriginal and Torres Strait Islander men's engagement with PHC is well established and compelling [2, 3, 4, 5, 6, 7, 8, 9, 10, 11, 12, 13, 14, 15, 16, 17, 18, 19, 20, 21, 22, 23, 24, 25, 26, 27, 28, 29, 30, 31, 32, 33, 34, 35, 36, 37, 38, 39, 40, 41, 42, 43, 44, 45, 46, 47, 48, 49, 50, 51, 52]. For more than a decade, research has documented barriers, enablers and effective program characteristics. Policy frameworks have acknowledged these findings, evaluations have confirmed them, and yet, systems and services remain largely unchanged [13, 14, 15, 32, 40, 49, 50].

Institutional risk aversion and short‐term political cycles impede progressive knowledge translation [11, 12, 23]. The gap between knowledge and action now constitutes structural neglect. The consistent commissioning of small‐scale pilot programs in the presence of distinct evidence laying bare the realities, constraints and recommendations faced by Aboriginal and Torres Strait Islander men, is the conscious avoidance of service and system reforms [14].

PHCS reorientation is no longer a technical challenge; it is an ethical and political imperative. If governments are genuinely serious about closing the health gap, reducing health inequities, improving life expectancy and reducing preventable disease burden among Aboriginal and Torres Strait Islander men, then system redesign must move from aspiration to obligation.

These persistent failures cannot be understood solely as technical or administrative shortcomings. They also reflect the ongoing effects of systemic and structural racism within Australian health systems, whereby Aboriginal and Torres Strait Islander priorities, knowledges and models of care continue to receive lower institutional value than mainstream approaches. The repeated failure to embed evidence‐based, community‐led models despite decades of consistent findings demonstrates that inequitable structures, rather than insufficient evidence, continue to shape policy and commissioning decisions. The evidence threshold has been met. What remains is the decision to act.

## Author Contributions

**Kootsy Canuto:** conceptualisation, writing – original draft, writing – review and editing and supervision. **Celina Gaweda:** writing – review and editing. **James Smith:** writing – review and editing. **Karla J. Canuto:** writing – original draft, writing – review and editing and supervision.

## Funding

Karla Canuto is funded by a National Health and Medical Research Council (NHMRC) 2025 Investigator Grant (APP2041519). Kootsy Canuto and Celina Gaweda are supported by the NHMRC 2025 Investigator Grant (APP2041519). James Smith is funded by an Australian Research Council Mid‐Career Industry Fellowship (IM250100083). Funding acknowledged herein supported researchers' salaries in reviewing the literature, analysing, interpreting and writing the manuscript. Publication costs were funded by the Australian Government Medical Research Future Fund (MRFF) – 2020 Primary Care Research grant scheme (APP2006564).

## Disclosure

Not commissioned; externally peer reviewed. Microsoft CoPilot was used to cross‐check references.

## Conflicts of Interest

The authors declare no conflicts of interest.

## Supporting information


**Data S1:** CONSIDER statement.

## Data Availability

This article includes no original data.
