# Supplementary material for: Beyond Structural Neglect: Using Evidence to Strengthen Primary Health Care Systems, Policies and Programs for Aboriginal and Torres Strait Islander Men
Source: Med J Aust. 2026 Oct 16;224(10):e70319. doi: 10.5694/mja2.70319 (PMC13675111; doi:10.5694/mja2.70319)
Supplement: Supplementary file 1 [file MJA2-224-0-s001.pdf]

The Medical Journal of Australia

Supporting Information

Supplementary material

This appendix was part of the submitted manuscript and has been peer reviewed.
It is posted as supplied by the authors.

Appendix to: Canuto K, Gaweda C, Smith J, Canuto KJ. Beyond Structural Neglect: Using Evidence to Strengthen Primary Health Care Systems, Policies and Programs for Aboriginal and Torres Strait Islander Men. *Med J Aust* 2026; doi: 10.5694/mja2.70319.

CONSIDER Statement Template

Guest Editors of the 2026 *Indigenous Health Special Issue* acknowledge the Indigenous expertise that informed the establishment of the CONSolIDated critERia for strengthening the reporting of health research involving Indigenous Peoples (CONSIDER) statement.

Authors should indicate how they have supported ethical publishing and reporting practices by providing the details of the research practices aligned with this publication in accordance with the CONSIDER statement. The reporting should not exceed two pages. This reporting will be published as online supplementary information. Detailed items can be accessed in the publication:

https://bmcmedresmethodol.biomedcentral.com/articles/10.1186/s12874-019-0815-8

**Governance**
No formal governance was convened for the purpose of writing this perspective piece. The extent of research was limited to drawing on existing literature and evidence to demonstrate the argument presented in the paper. The concept, progression and structure of the writing in this paper was conducted by two Indigenous lead authors.
**Prioritization**
The lead author, Associate Professor Kootsy Canuto's incentive for writing this paper comes from over a decade working in Aboriginal and Torres Strait Islander men's health research – with a particular lens on primary health engagement. His extensive research highlights the ongoing systemic failure to adequately address issues in Aboriginal and Torres Strait Islander men's health despite repeated and consistent evidence.
**Relationships (Indigenous stakeholders/participants and Research Team**
The authors of this article include two Torres Strait Islander and two non-Indigenous researchers. They have worked together as researchers on multiple Aboriginal and Torres Strait Islander health projects over many years. The relationships are built and maintained on trust, integrity and transparency. As a team, they pride themselves on the way they conduct research that addresses community identified priorities that are governed and driven by Aboriginal and Torres Strait Islander researchers collaboratively with Aboriginal and Torres Strait Islander people.
**Methodologies**
Written through an Indigenous lens, this perspective piece prioritises the lead author's Associate Professor Kootsy Canuto's relationality, lived experience, and positionality as a Torres Strait Islander male.
**Participation**
Not applicable. No human participants were involved in the research for writing this article.
**Capacity**
Not applicable. The Indigenous authors of this article are academic professionals.
**Analysis and interpretation**
Not applicable. No qualitative or quantitative data was collected from participants or analysed or interpreted for writing this article.
**Dissemination**
Not applicable. As above.
